# The Medical Application of Electro-Magnetism

**Published:** 1854-02

**Authors:** 


*The Medical Application of Electro-Magnetism.* By SAMUEL B. SMITH.

This is a substantial pamphlet, devoted to the laudation of Mr. Smith's electro-magnetic machine. The advertising is alone incidentally, and is interspersed with quotations from Matteucci and sundry other experimenters. It of course adopts the hypothesis that nerve-force and electricity are identical. We cannot commend the doctrines of the book, though we cheerfully acknowledge the ingenuity of the advertisement. If equal Yankee skill is displayed in the machine itself, it is, doubtless, a very good one. H.

